# The leaf-mining genus *Antispila* Hübner, 1825 feeding on Vitaceae in Shandong Peninsula, China with one new species (Lepidoptera, Heliozelidae)

**DOI:** 10.3897/zookeys.744.22421

**Published:** 2018-03-19

**Authors:** Nan Wang, Tengteng Liu, Jiasheng Xu, Bin Jiang

**Affiliations:** 1 Shandong Provincial Key Laboratory of Animal Resistance Biology, College of Life Sciences, Shandong Normal University, Jinan 250014, P. R. China; 2 School of Life and Environmental Science, Gannan Normal University, Ganzhou 341000, P. R. China; 3 Kunyushan Forest Farm, Yantai 264002, P. R. China; 4 Kunyushan National Nature Reverse, Yantai 264002, P. R. China

**Keywords:** *Ampelopsis*, *Antispila*, China, DNA barcode, Heliozelidae, Lepidoptera, new species, Shandong Peninsula, *Vitis*

## Abstract

The *Antispila* species feeding on Vitaceae from Shandong Peninsula, China are treated. *Antispila kunyuensis* Liu, **sp. n.**, feeding on *Ampelopsis humulifolia*, is described as new to science, and *A. uenoi* Kuroko, 1987, feeding on *Vitis amurensis*, is recorded as new for China. *Vitis amurensis* is documented as a new host plant for *A. uenoi*. The adult male and female, host plant and typical patterns of leaf-mines of both species are illustrated, as are male and female genitalia and venation. The venation and the paired tufts of scales on the 7^th^ abdominal segment in male are illustrated for *A. uenoi* for the first time. DNA barcodes of both species are also provided, together with a neighbor-joining tree for facilitating species delimitation.

## Introduction

The family Heliozelidae comprises 126 described species in 12 genera (van Nieukerken et al. 2011, 2012, van Nieukerken and Geertsema 2015, Liu and Wang 2017), with the largest diversity in North America and Australia. The Heliozelidae were only recently recorded as new for China, with one formally published species (Liu and Wang 2017), but also several database records in BOLD (http://www.boldsystems.org). However, the knowledge of Chinese Heliozelidae is slowly increasing, and two more species feeding on Vitaceae are described in the present paper. Vitaceae comprises an important group of hosts for Heliozelidae, especially for the genus *Antispila* Hübner, 1825 (van Nieukerken et al. 2012). Eleven out of the 20 Palearctic and Oriental species of *Antispila* feed on Vitaceae (Meyrick 1926, Kuroko 1961, 1987, van Nieukerken et al. 2012, van Nieukerken and Geertsema 2015).

Shandong Peninsula is located in the east of China, facing the Korean Peninsula across the Yellow Sea. Although located in a relatively developed region, the arthropod diversity is still rather poorly known and lacks systematic work, so new species, especially small-sized ones, can still be discovered. Here, we discovered two species of the leaf-mining genus *Antispila* during an ongoing biodiversity exploration in Shandong Peninsula. One of these is a new species, the other one, *A. uenoi* Kuroko, 1987 is new for China. Both are described here in detail, increasing the number of known species of the genus *Antispila* in China to three.

## Material and methods

Leaves with active mines were placed in small plastic bags for rearing. After the shields had been exscinded and the larvae had left the mines, leaves with vacant leaf-mines were dried in a plant press. The larval shields, the corresponding adults, and the vacant leaf-mines were identically coded.

Genitalia and wings were dissected and mounted according to the methods introduced by Li (2002), but stained with Eosin Y and/or Chlorazol Black. Illustrations were prepared by using a Leica DM1000 microscope. Adult photographs were taken with a Leica S6D stereo microscope. Photographs of the host plants and leaf-mines were taken in the field using a Canon EOS 650D camera plus a Macro Lens, and enlarged photographs of leaf-mines were taken with the Leica S6D stereo microscope.

DNA was extracted from adult specimens preserved in 95% ethanol in Shandong Normal University, Jinan, China, with the whole body skeleton including genitalia and wings preserved as vouchers (Knölke et al. 2005). Protocols for total DNA extraction and mitochondrial COI gene amplification followed that described in our previous study (Liu and Wang 2017). The sequence data used in this study have been deposited in GenBank and in the BOLD database, a public dataset “DS-ANTIVIT” (https://doi.org/10.5883/DS-ANTIVIT). Sequences were aligned using the MUSCLE model and genetic distance estimation was analyzed using the Kimura 2-Parameter model in BOLD.

Terminology for adults follows van Nieukerken et al. (2012); the term canalis spiralis of female genitalia follows Kuroko (1987). The classification of the host plants is based on APG (2016), and plant scientific names follow The Plant List (2013).

All the specimens examined, including the holotype of the new species, are deposited in the Zoological Collection of Shandong Normal University (**SDNU**). The type depository of *A. uenoi* Kuroko, 1987, collection of the Entomological Laboratory, University of Osaka Prefecture, is abbreviated as **UOP**.

## Taxonomy

### Antispila kunyuensis

Taxon classificationAnimaliaLepidopteraHeliozelidae

Liu sp. n.

http://zoobank.org/25296AE0-2076-4A19-913B-617825812FF4

Figs 1 , 2 , 6 , 9 , 11–15 , 26–29 , 34–38

#### Type material.

**Holotype**. ♂, China, Shandong Province, Yantai, Mt. Kunyu National Nature Reverse, 121.740°E, 37.292°N, 400 m, larva coll. 2017.vi.28, mine on *Ampelopsis humulifolia*, emerged viii.06, collector Bin Jiang, genitalia no. SDNU.LIU0014, registered no. SDNU.YT170601.2. **Paratypes**. 3♂, 3♀, genitalia nos. SDNU.LIU0009♂, SDNU.LIU0016♀, SDNU.LIU0044♂, DNA voucher slide no. SDNU.LIU0013♀ (whole body on one slide), registered nos. SDNU.YT170601.1, SDNU.YT170601.3–6, other data same as holotype.

#### Other material.

**Leaf-mine.** Shandong Province: Yantai, Mt. Kunyu National Nature Reverse, 121.740°E, 37.292°N, 400 m, vacant mine coll. 2016.vii.31, on *Ampelopsis humulifolia*, collector Tengteng Liu & Encui Wang, registered no. SDNU.YT160761–7, YT160785.

#### Diagnosis.

Two *Antispila* species, *A. ampelopsia* Kuroko, 1961 and *A. orbiculella* Kuroko, 1961, are known to feed on *Ampelopsis*, and both associate with the same species, *A. brevipedunculata*. *Antispila kunyuensis* can easily be distinguished from *A. ampelopsia* by the fine features of the phallus and the ovipositor, and from *A. orbiculella* by the two separate basal spots that are joined forming a transverse fascia in the forewing.

#### Description.

**Figures 1–8.**
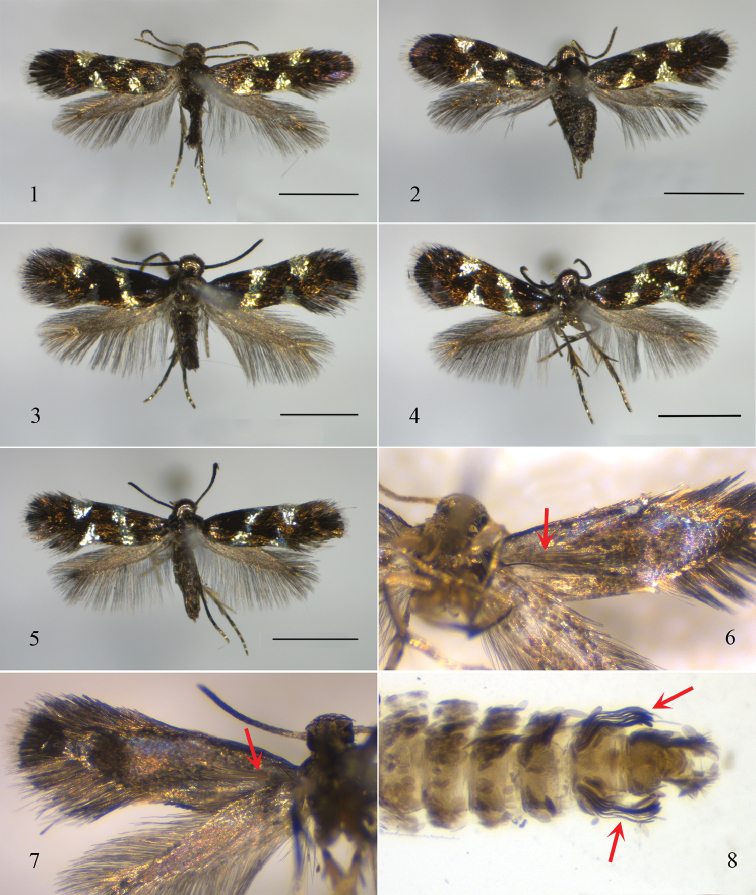
Adult of *Antispila* species. **1** *A. kunyuensis*, holotype, male, SDNU.QD170705.1 **2** *A. kunyuensis* sp. n., paratype, female, SDNU.YT170601.4 **3** *A. uenoi*, male, SDNU.QD170705.1 **4** *A. uenoi*, female, SDNU.QD170705.3 **5** *A. uenoi*, female, SDNU.QD170707 **6** *A. kunyuensis*, male, paratype, ventral view of forewing indicating bristles of retinaculum, SDNU.YT170601.3 **7** *A. uenoi*, ventral view of forewing indicating bristles of retinaculum **8** *A. uenoi*, male, indicating paired tufts of slender scales on the 7^th^ abdominal segment, photographed during dissection in water, SDNU.QD170705.1. Scale bars: 1.0 mm.

**Adult** (Figs 1, 2, 6). Forewing length 1.7–2.1 mm. Head silvery gray, with reddish reflection, more apparent on front. Antennae dark fuscous, silvery gray on distal two segments. Labial palpus silvery gray, pointed apically. Thorax and tegula dark fuscous. Legs gray, with blackish gray pigmentation outer surface. Forewing dark fuscous, with strong reddish reflection; two pairs of opposite triangular silvery spots on costa and dorsum, the inner pair with costal spot before middle, dorsal spot at basal 1/4, the outer pair with costal spot at 3/4, dorsal spot largest, near tornus; cilia unicolorous with forewing on basal 3/4, whitish gray on distal 1/4. Hind wing dark gray, cilia darker. Abdomen dark gray dorsally, yellowish gray ventrally.

**Figures 9–10.**
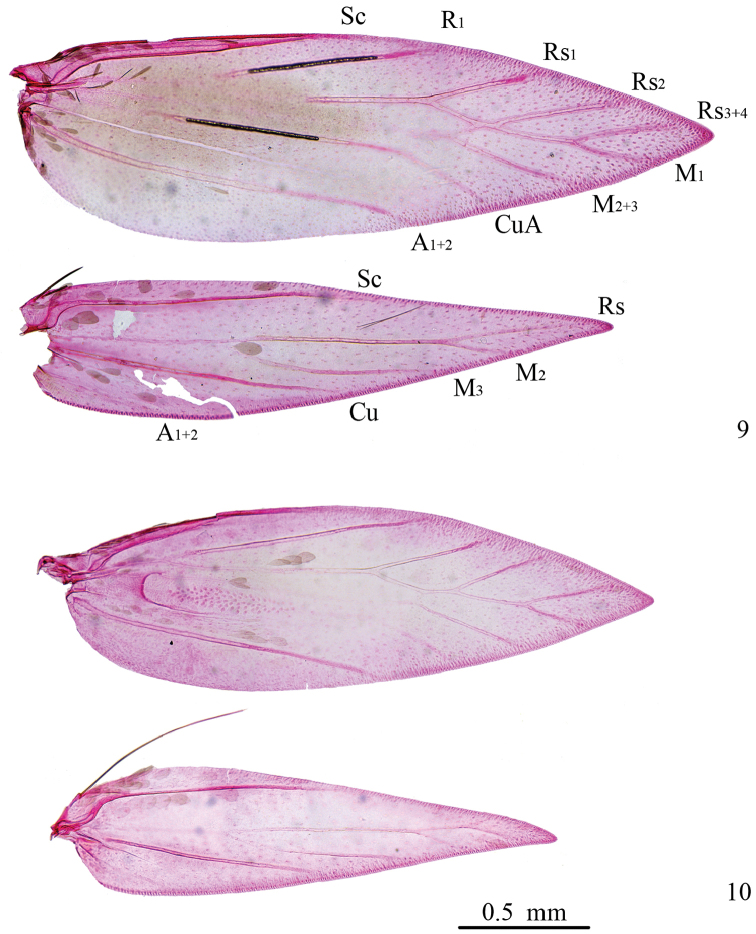
Wing venation of *Antispila* species. **9** *A. kunyuensis*, paratype, female, SDNU.LIU0013 **10** *A. uenoi*, male, SDNU.LIU0012.

*Venation* (Fig. 9). Forewing with Sc reaching before middle on costa; R_1_ from 2/5 on upper margin of cell to costal 3/5, Rs_1_ from distal 1/7 on upper margin of cell to costal 3/4, Rs_2_ from beyond distal end of cell, Rs_3+4_ reaching costa before apex; cell triangular distally; M_1_ stalked with Rs_3+4_, to termen near apex, M_2+3_ from near distal end of cell; CuA from distal 1/6 of lower margin of cell; A_1+2_ to beyond middle of dorsum. Hindwing with Sc to costal 3/5, R+M ending in 3 branches: Rs to costa near apex, M_2_ and M_3_ to dorsum; Cu to middle of dorsum; A_1+2_ weak. Male with one long frenulum, female bearing two shorter frenular bristles.

**Figures 11–15.**
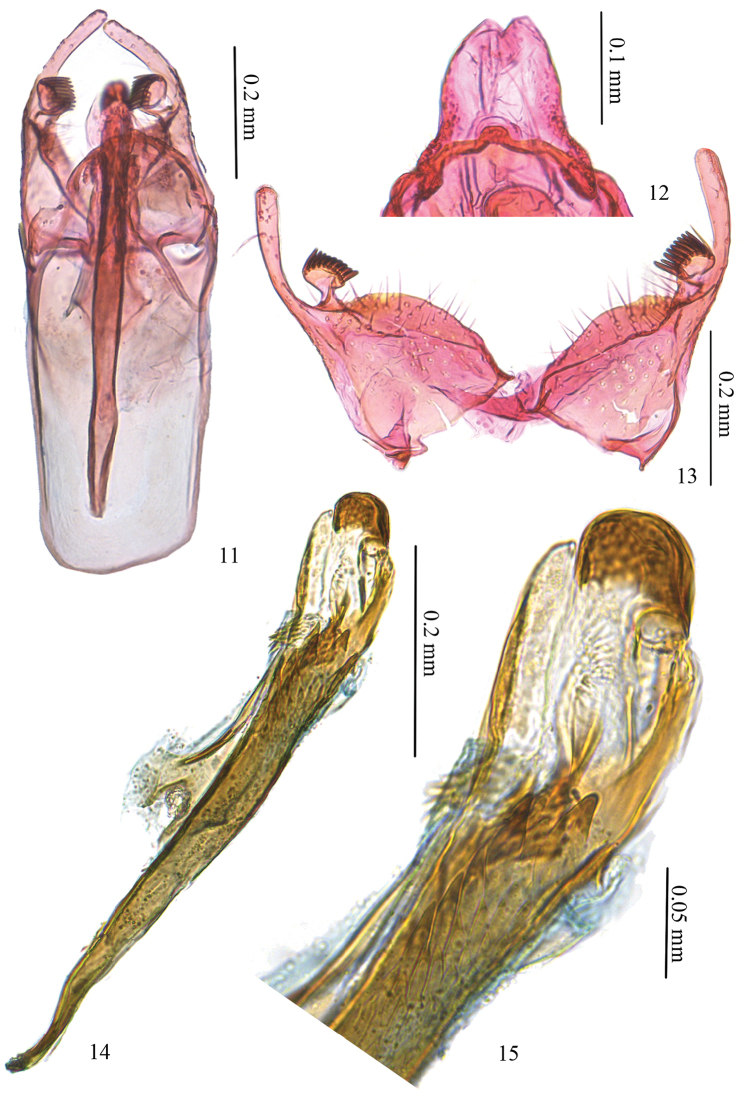
Male genitalia of *Antispila kunyuensis*. **11** whole genitalia, SDNU.LIU0014 **12** tegumen, SDNU.LIU0009 **13** valva, same slide as 12 **14** phallus, lateral view, SDNU.LIU0044 **15** enlarged distal part of phallus, same slide as 14.

*Male genitalia* (Figs 11–15). Tuba analis developed (Fig. 12). Uncus bar-shaped, protruded towards posteriorly at middle. Vinculum shorter than phallus, slightly rectangular on anterior margin. Valva more or less triangular, digital process long and narrow, almost same length as valva, pecten on pedicel, with nine comb teeth (Fig. 13). Juxta half as long as phallus, densely covered with small teeth on basal 2/5, anterior arrow pointed on basal corners. Phallus nearly as long as length of vinculum + tegumen, narrowed anteriorly (Fig. 11); phallotheca with five to six strong teeth and a group of smaller sharp-pointed teeth, with a cluster of smaller spines at base of juxta; distal part with a mushroom-shaped process and a straight process ventrally (Figs 14, 15).

*Female genitalia* (Figs 26–29). Ovipositor with three cusps at either side, with basal one largest and middle one smallest (Fig. 28). Vestibulum round, more or less sclerotized (Fig. 29). Corpus bursae membranous.

#### DNA barcode.

One DNA barcode from a paratype was obtained. A neighbor-joining tree, covering most Asian *Antispila* species and other Vitaceae-feeding species, was generated for facilitating species delimitation (Fig. 33).

#### Host plant.

*Ampelopsis humulifolia* Bunge (Figs 34, 35). The placement of the leaf-mines is variable (Figs 36–38), from the base to the apex of a leaf, from absolutely along veins to between but not touching veins. This results in variably-shaped blotch mines, but the majority of blotches are more or less round (Figs 38, 42–45). Frass primarily occupies the opposite side of the cut-out in round blotch mines, or occasionally disperses throughout the mine (Fig. 37), but always in a broad medial band in wide gallery mines (Fig. 38). This species overwinters as a prepupa in the shield. A single generation per year was observed at the type locality.

#### Distribution.

China (Shandong).

#### Etymology.

The specific name is derived from the type locality of the new species, Mt. Kunyu, representing the most famous nature reserve in Shandong Peninsula, focusing on forest ecosystem conservation.

### Antispila uenoi

Taxon classificationAnimaliaLepidopteraHeliozelidae

Kuroko, 1987

Figs 3–5 , 7 , 8 , 10 , 16–18 , 19–25 , 30–31 , 39–41 , 46–49

Antispila uenoi Kuroko, 1987: 113. TL: Japan (Iwate Prefecture). TD: UOP.

#### Material examined.

China: 2♂, 3♀, Mt. Laoshan, Qingdao, Shandong Province, 120.609°E, 36.204°N, 400 m, larva coll. 2017.vii.01, mine on leaf serration of *Vitis amurensis*, case made vii.03, emerged vii.15, collectors Tengteng Liu and Nan Wang, genitalia no. SDNU.LIU0008♀, SDNU.LIU0015♂, DNA voucher slide no. SDNU.LIU0011♂ (whole body on one slide), registered no. SDNU.QD170705.1–3; 2♂, 2♀, Mt. Laoshan, Qingdao, Shandong Province, 120.609°E, 36.204°N, 400 m, larva coll. 2017.vii.01, mine on leaf basal area, case made vii.03, emerged vii.15, genitalia no. SDNU.LIU0043♀, DNA voucher slide no. SDNU.LIU0012♂ (whole body on one slide), collectors Tengteng Liu and Zhenquan Gao, registered nos. SDNU.QD170707, SDNU.QD1707.1–2.

#### Diagnosis.

Kuroko (1987) gave a detailed diagnosis to distinguish *A. uenoi* from *A. ampelopsia*.

#### Description.

*Adult* (Figs 3–5, 7). Forewing length 1.6–1.8 mm. Head silvery gray, with reddish and purple reflection. Antennae dark fuscous, silvery on distal two segments. Labial palpus silvery gray, pointed apically. Thorax and tegula dark fuscous. Legs black, with whitish gray pigmentation on distal part of tarsomeres. Forewing blackish fuscous, with strong purple reflection; an oblique silvery fascia from before middle on costa to basal 1/4 on dorsum, a triangular silvery spot on costal 3/4, with a similar one opposite to it near tornus; cilia unicolorous with forewing on basal 3/4, whitish gray on distal 1/4. Hind wing gray, cilia darker. Abdomen dark gray dorsally, gray ventrally.

*Female* with forewing patterns more distinct (Figs 4, 5).

***Venation*** (Fig. 10). Forewing with Sc reaching before middle on costa; R_1_ from 2/5 on upper margin of cell to costal 3/5, Rs_1_ from distal 1/7 on upper margin of cell to costal 3/4, Rs_2_ from well beyond distal end of cell, Rs_3+4_ reaching costa before apex; cell truncated distally; M_1_ stalked with Rs_3+4_, to termen near apex, M_2+3_ from lower corner of distal end of cell; CuA from distal 1/7 of lower margin of cell; A_1+2_ to beyond middle of dorsum. Hindwing with Sc to beyond middle of costa, R+M ending in 4 branches: Rs to costa, M_1_ to dorsum near apex, M_2_ and M_3_ to dorsum; Cu to middle of dorsum; A_1+2_ weak. Male with one long frenulum, female bearing two shorter frenular bristles.

**Figures 16–18.**
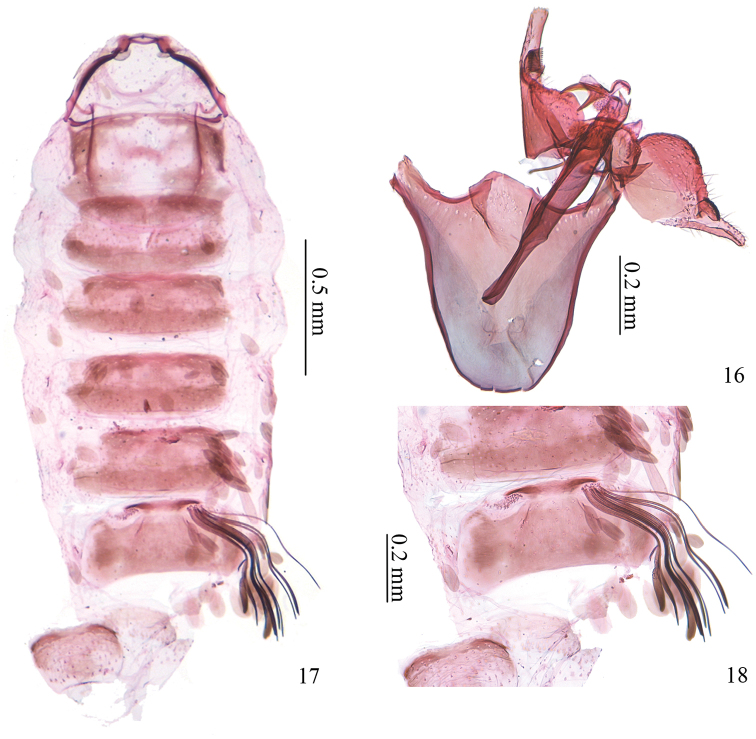
Male genitalia and abdomen of *Antispila uenoi*. **16** whole male genitalia, unrolled, SDNU.LIU0015 **17** abdomen, ventral view, notice the tuft of slender scales on the 7^th^ segment, left tuft detached, same slide as 16 **18** enlarged view of tuft of slender scales, same slide as 16.

**Figures 19–25.**
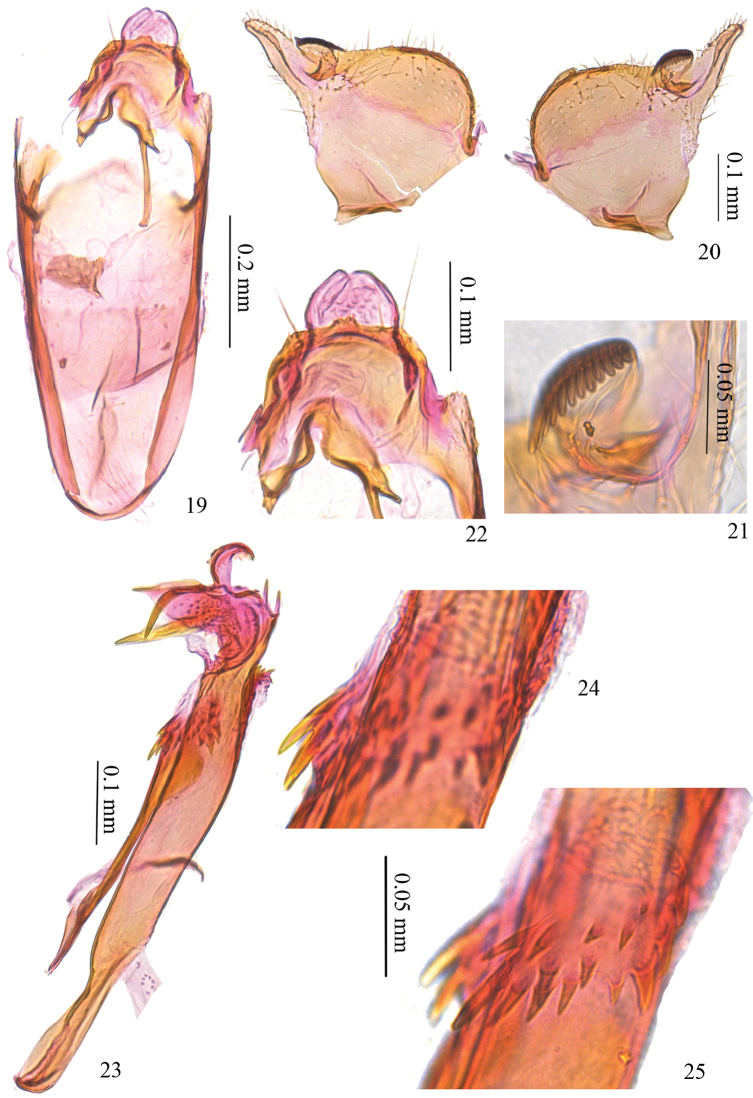
Male genitalia of *Antispila uenoi*. **19–23** SDNU.LIU0011 **19** tegumen and vinculum, **20** valva **21** enlarged view of comb **22** tegumen **23** phallus, lateral view **24** enlarged view of spines on base of juxta, SDNU.LIU0012 **25** same part as 24 under a shallow focus.

*Male genitalia* (Figs 16–25). Tuba analis developed. Uncus bar-shaped, with two papillae bearing two short setae each at middle, bearing one long and a few shorter setae laterally (Fig. 22). Vinculum shorter than phallus, rounded on anterior margin. Valva semicircular on ventral margin, digital process about half the width of valva, pecten with 12 comb teeth (Figs 20, 21). Juxta longer than half length of phallus, anterior arrow broad and almost semicircular. Phallus as long as length of vinculum + tegumen, narrowed anteriorly; phallotheca with groups of spines, more concentrated and larger ventrally (Figs 24, 25); distal part with two processes ventrally, one large and curved, the other V-shaped with one branch larger than the other, two smaller similar processes dorso-apically, one less sclerotized and curved process at apex with several membranous teeth ventrally (Fig. 23). Paired tufts of slender scales on the 7^th^ abdominal segment (Figs 8, 17, 18).

**Figures 26–29.**
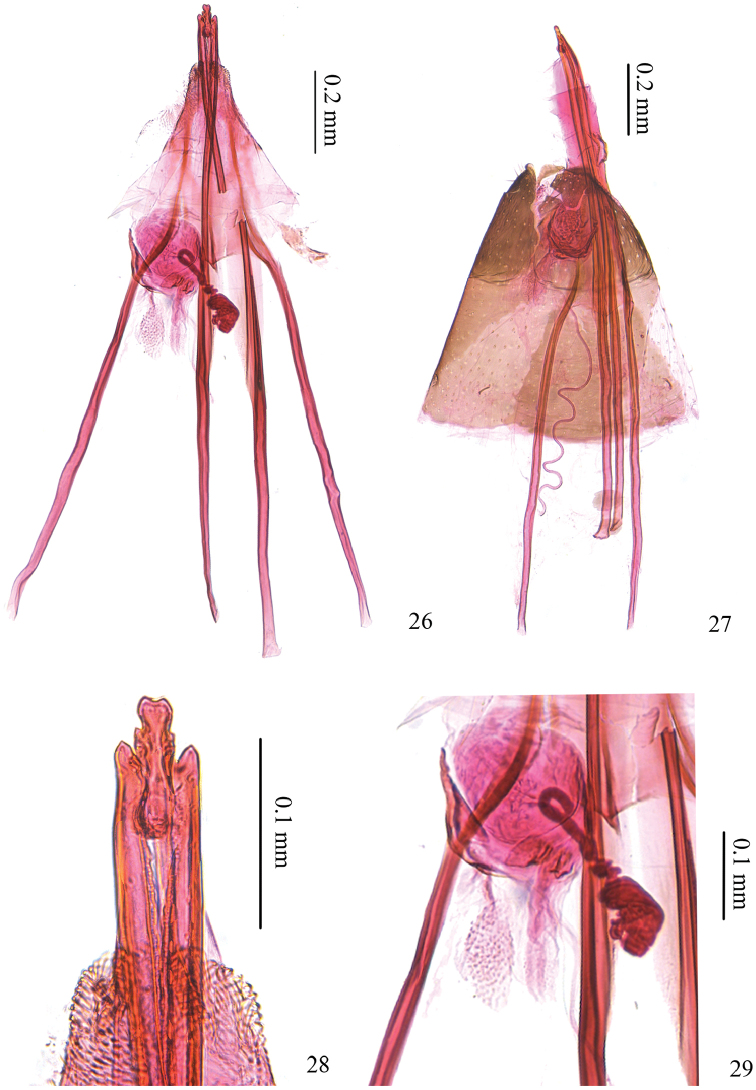
Female genitalia of *Antispila kunyuensis*. **26** female genitalia, SDNU.LIU0016 **27** female genitalia, SDNU.LIU0013 **28** detail of ovipositor tip, same slide as 26 **29** detail of vestibulum, same slide as 26.

**Figures 30–32.**
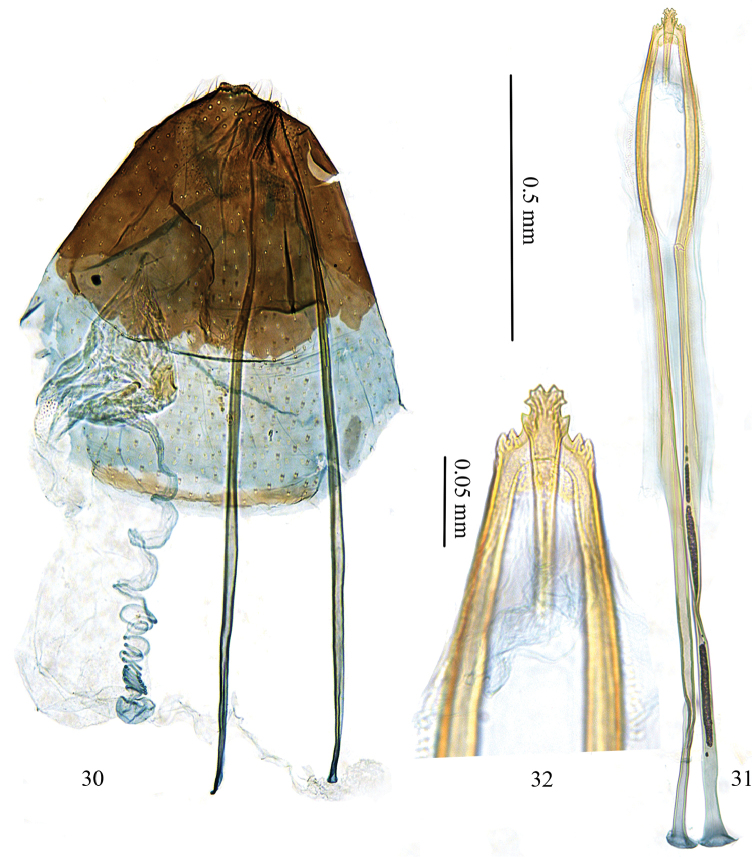
Female genitalia of *Antispila uenoi*. **30** female genitalia, SDNU.LIU0043 **31** posterior apophyses and ovipositor tip, same slide as 30 **32** detail of ovipositor tip, same slide as 30.

*Female genitalia* (Figs 30–32). Ovipositor with six cusps at either side, with apical three smaller, tip distinctly indented (Fig. 32). Vestibulum membranous, with a sclerotized granule and a weak circular ring surrounding opening of canalis spiralis (Fig. 30). Corpus bursae membranous.

#### DNA barcode.

**Figure 33.**
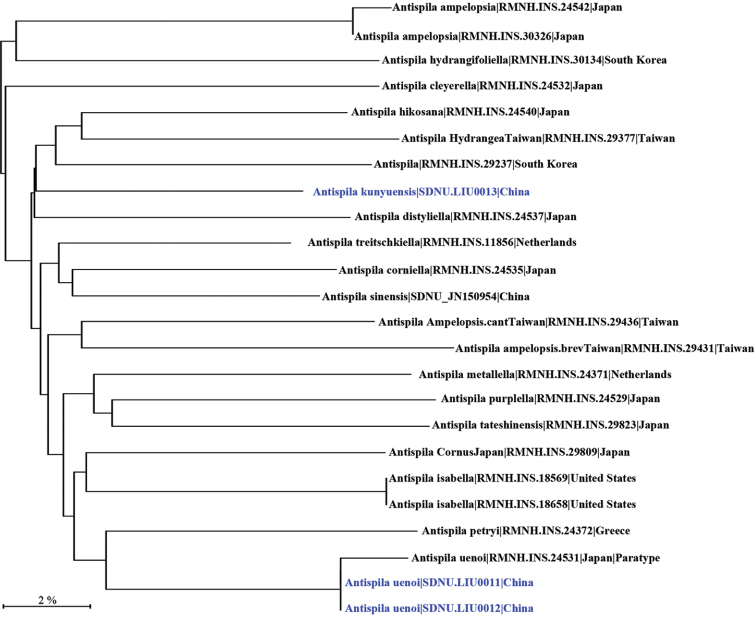
Neighbor Joining Tree, based on DNA barcodes of *Antispila* species, especially Vitaceae-feeding and Asian species.

Two DNA barcodes were obtained (Fig. 33). A partial DNA barcode of 268 bp generated from a paratype of *A. uenoi* (RMNH.INS.24531) was used for identification of the Chinese specimens. The genetic distance between the Chinese specimens and the paratype is 1.53%.

#### Host plants.

*Vitis amurensis* Rupr. (Fig. 39), *V. coignetiae* Pulliat ex Planch. and *V. labruscana* L.H. Bailey (Kuroko 1987). *Vitis amurensis* is recorded here as a new host.

#### Biology.

Leaf-mines on *Vitis amurensis* can occupy serrations along the leaf margin (Figs 40, 41, 46–48) or the leaf basal area (Fig. 49) in an almost equal proportion, calculated from our rearing data (5 : 4); no other placements (e. g. leaf central area) of mines were observed, although in Japan the majority of mines occupy the apical or marginal area of the leaves on other hosts (Kuroko 1987). Frass often dispersed along mines (Figs 47–49). This species overwinters as a prepupa in the shield. Two generations probably occur in Shandong Peninsula.

**Figures 34–41.**
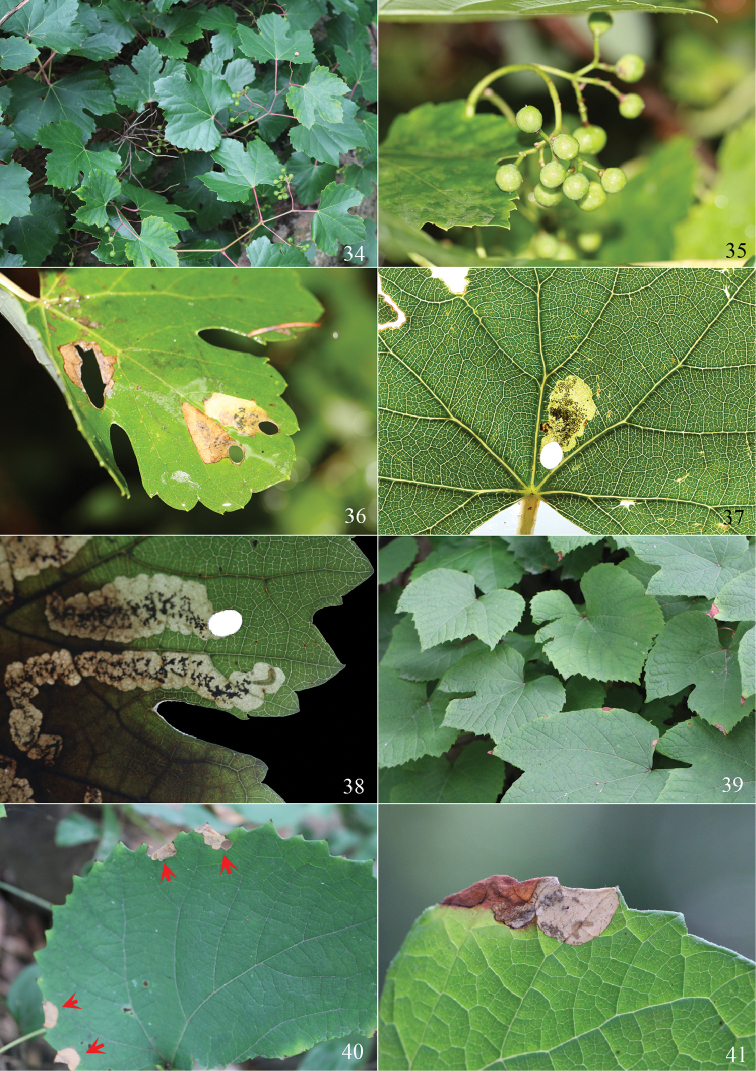
Host plant and leaf-mines of *Antispila* species. **34–38** *A. kunyuensis* **34** Host plant, *Ampelopsis humulifolia* **35** unripe fruits of *A. humulifolia* **36–38** leaf-mines **39–41** *A. uenoi* **39** Host plant, *Vitis amurensis* **40** leaf-mines along leaf margin, indicated by red arrows **41** leaf-mine with a dead larva.

**Figures 42–49.**
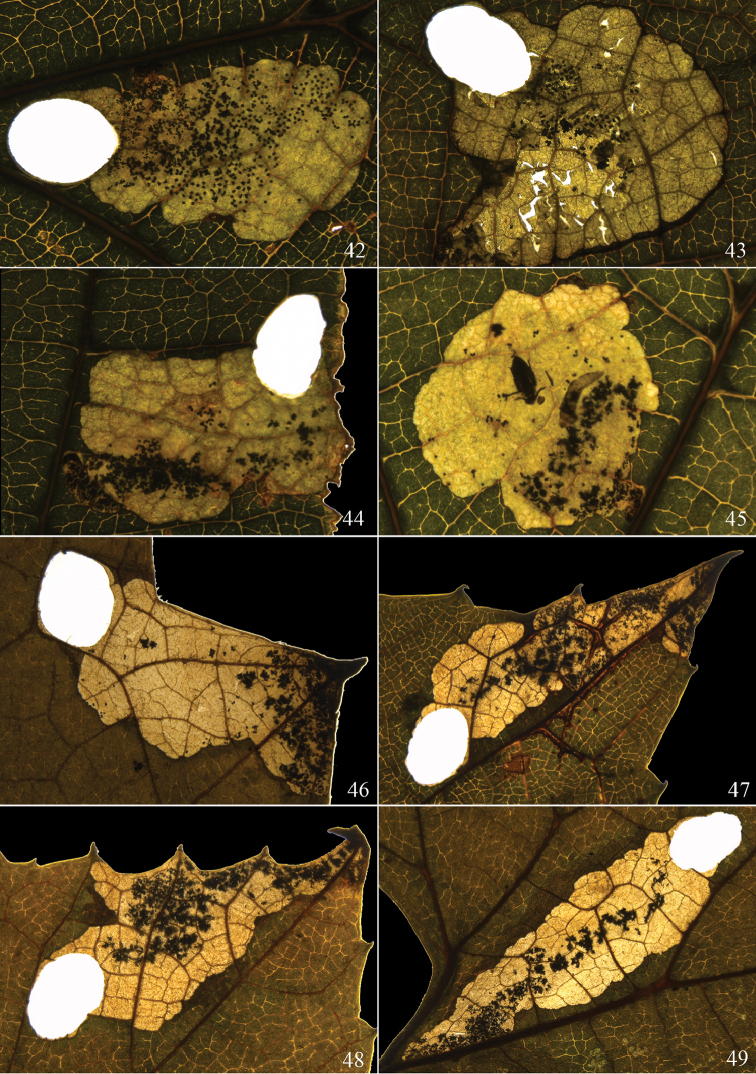
Leaf-mines of *Antispila* species. **42–45** *A. kunyuensis*, fig. **42** is the identical mine to Fig. 37, one dead larva and a parasitoid, most likely a species of Eulophidae in Figure 45 **46–49** *A. uenoi*, leaf-mines occupying leaf serration and apical area in Figures 46–48, leaf-mine occupying leaf basal area in Figure 49.

#### Distribution.

China (Shandong), Japan: Honshu. The host plant *Vitis amurensis* is widespread in the northeast and eastern parts of China (Chen et al. 2007), Eastern Russia (Afonin et al. 2008), Japan: Honshu and Korea (Ohwi 1965). A much wider distribution of the moth is expected, where its host plants occur.

#### Remarks.

This species is newly recorded in China. The venation and the paired tufts of scales on the 7^th^ abdominal segment in male are illustrated for *A. uenoi* for the first time.

## Discussion

In the Miocene, the arthropod diversity of Shandong Peninsula was quite rich, which is well documented by numerous fossil records from Shanwang, Shandong province (Zhang 1989, 1994). During the last centuries, the Yellow River shifted its mouth a number of times and finally diverted to the Bohai Sea in 1855 (Cheng and Xue 1997, Wang et al. 2010). Both long-term historical and recent regional processes may have significantly influenced the species richness of Shandong Peninsula, which makes this peninsula an interesting region for biogeographical and biodiversity studies (e.g., Zheng et al. 2009). Heliozelidae, together with other small-sized Lepidoptera species, are expected to experience an increase in species richness upon a deeper exploration of this region, as will be the case for all of China. For instance, in Argyresthiidae, the number of species increased from 14 to 64 after the study by Liu et al. (2017). This would be true when considering the host preference of the heliozelids and the rich diversity of Vitaceae and many other host families in China. Take Vitaceae for example. A majority of *Antispila* species show a specific host preferences for Vitaceae and Cornaceae (Milla et al. 2017, van Nieukerken et al. 2018), and there are 146 species of Vitaceae distributed in eight genera in China, with 87 endemic species, which are mostly native to central, south, and southwest China (Chen et al. 2007).

## Supplementary Material

XML Treatment for Antispila kunyuensis

XML Treatment for Antispila uenoi
